# Endocytosis and transcytosis of gliadin peptides

**DOI:** 10.1186/s40348-015-0029-z

**Published:** 2016-02-16

**Authors:** M. Vittoria Barone, K. Peter Zimmer

**Affiliations:** Department of Translational Medical Science, University of Naples, Federico II, Via S. Pansini 5, 80131 Naples, Italy; ELFID (European Laboratory For the Investigation of Food Induced Disease), University of Naples, Federico II, Via S. Pansini 5, 80131 Naples, Italy; Children’s Hospital, Justus Liebig University, Feulgenstr. 12, 35392 Gießen, Germany

**Keywords:** Celiac disease, Gliadin peptides, Peptide 31–43 (P31–43), Endocytosis, Transcytosis, EEA1, LAMP

## Abstract

**Background:**

Celiac disease (CD) is a frequent inflammatory intestinal disease, with a genetic background, caused by gliadin-containing food. Some gliadin peptides are not digested by intestinal proteases and can have different biological effects. Gliadin peptides can induce innate and adaptive T cell-mediated immune responses. The major mediator of the stress and innate immune response to gliadin peptides (i.e., peptides 31–43 and 31–55) is the cytokine interleukin-15 (IL-15). Other peptides such as the 33 mer containing the P57–68 sequence, after tissue transglutaminase deamidation, are well presented to T cell in the intestine and can induce an adaptive immune response.

**Findings:**

In this paper, we review the recent studies on the digestion of gliadin and the peptides released by the digestion process. We will also discuss the mechanisms responsible for the internalization and transcytosis of indigested gliadin peptides in the intestinal epithelium.

**Conclusions:**

Gliadin is not completely digested by the intestinal proteases producing bioactive peptides that have different biological effects. These peptides are internalized in the cells by an active process of endocytosis and can traverse the intestinal mucosa with different kinetics and immunological effects. In vivo findings will also be discussed.

## Background

Celiac disease (CD) is a common enteropathy induced by ingestion of wheat gluten proteins and related prolamins from oat, rye, and barley in genetically susceptible individuals. The genetic predisposition is characterized by the presence of human leucocytes antigen (HLA)-DQ2 and HLA-DQ8 molecules in virtually all CD patients [1]. The main toxic components of gluten belong to a family of closely related proline- and glutamine-rich proteins called gliadin. This is a heterogeneous mixture of proteins that can be assigned to three major groups (i.e., α/β-, γ-, and ω-gliadins) [2]. The high percentage of proline residues makes gliadin resistant to gastric–pancreatic and intestinal digestive proteases, so that long gliadin fragments can reach high concentration levels in the gut epithelium [3, 4].

To date, a very high number of gluten peptides, deriving from α- and γ-gliadins, and recently from glutenins, have been reported to stimulate cluster of differentiation 4 (CD4)+ T lymphocytes isolated from the small intestinal mucosa of CD patients [1]. Although at least 50 T cell stimulatory gluten epitopes in native or deamidated form have been identified, the 33-mer peptide is considered the most immunogenic including six overlapping epitopes. The 33-mer peptide, released from recombinant α2-gliadin (Swiss-Prot accession number: Q9M4L6) through gastric–pancreatic enzyme digestion, is highly resistant to further digestion by intestinal brush border enzymes (brush border membranes (BBM)) [5]. Hence, it has been suggested that the 33-mer could reach the underlying lamina propria (LP) and, following deamidation, can play a central role in the pathogenic cascade of CD by activating the adaptive immune response.

More recently, attention has been directed to the possible involvement of innate immune mechanisms in CD [4]. In particular, it has been showed that the synthetic peptide α-gliadin 31–43 (LGQQQPFPPQQPY) is able to up-regulate the expressions of interleukin (IL)-15 on CaCo-2 cells surface-induced epithelial cell proliferation and IL-6 production [4]. The sequence 31–43 is comprised of a larger peptide 25 mer (P31–55) together with the slightly longer P31–49 have been deeply studied in the last years [4].

These observations indicate that in addition to its interactions with dendritic cells and T lymphocytes of the lamina propria, gliadin has a dual effect on the intestinal epithelium of CD patients, both activating the innate and the adaptive immune response through a different set of gliadin peptides.

In the present study, we will review the recent literature about how these active gliadin peptides can be generated by digestion and mainly how they are transported in the enterocytes and through the epithelium in vitro and ex vivo systems.

### Gliadin peptides digestion

**Fig. 1:**
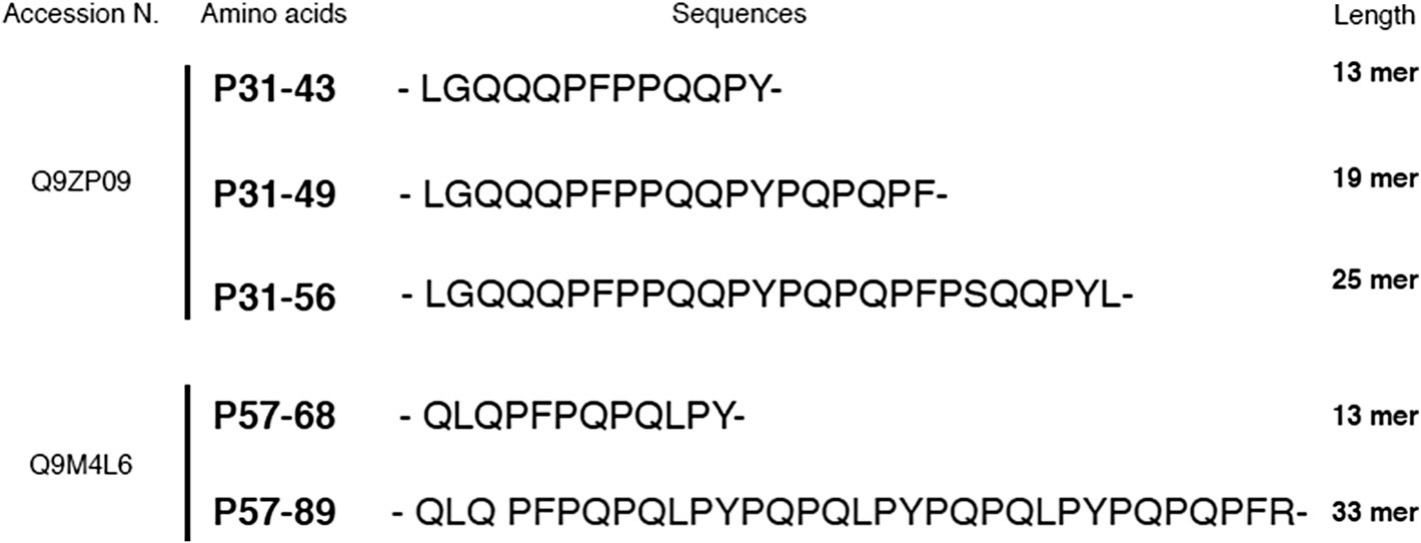
Sequences comparison of gliadin peptides. Twenty-five and 33 mer were the gliadin peptides most resistant to intestinal peptides. Swiss-Prot accession number, amino acids, and length were shown

Gliadin is a protein difficult to digest [5]. Several studies have approached the issue of gliadin digestibility in different systems. Recent studies have considered the digestion of a recombinant α-gliadin Q9ZP09. The in vitro digests of the recombinant protein produced a number of peptides, but supplementation with BBM greatly simplified the digests leaving two main peptides, the 25-mer peptide 31–55 and the 33-mer. Incubation of the synthetic 25 mer with BBM enzymes for longer reaction times confirmed its high resistance to proteolytic enzymes. Surprisingly, in the same system, the immuno-active epitope for the adaptive T cell-mediated immune response (P56–68), also included within gliadin Q9ZP09 sequence, was partly digested by gastric/pancreatic enzymes and completely digested after BBM supplementation. Indicating that gliadin Q9ZP09 was extensively hydrolyzed in vitro while the peptide sequence stimulating the innate response (P31–55) in CD was unaffected [6]. More recently, the same group analyzed the digestion of common pasta (*Triticum durum* semolina), using a sophisticated in vitro multi-compartment model that included oral, gastric, and duodenal phases of digestion [6]. Interestingly, the digestion of the cooked pasta showed the persistence of several wheat-derived peptides identified by liquid chromatography–mass spectrometry that included a-gliadin 31–55 and the shortened form a-gliadin 31–43 (Fig. 1). These studies demonstrate that these gliadin peptides can really come into contact with the intestinal epithelium in our everyday life and that in vitro studies using them have a physiologic rationale. Moreover, these data indicate that digestion of the P31–43 (or -49 or 25-mer) sequence is very difficult, implying that its biological activity can be central to the CD pathogenesis. An immediate application of these observation would be to find cereals that are deprived of the most indigested peptides to use in the CD patients diet or to find ways to process gluten-containing produces as to render them deprived of the host-indigested peptides.

### Gliadin peptides P31–43 and P57–68 enter the cells by an active process

Gliding peptides enter the cells by endocytosis. In fact, their entrance into the cells requires 37 °C temperature and Ca++ in the media [7]. Experiments with an inhibitor of endocytosis (methyl-β-cyclodextrin, M-β-CD) reduced the entrance of P31–43 labeled with a fluorescent tail such as lissamine (liss). Interestingly, the entrance of P31–43-liss was unaffected by filipin, an inhibitor of lipid raft/caveolae-mediated endocytosis. The opposite effect was generated by these inhibitors on P57–68-liss, indicating that both peptides enter intestinal epithelial cells by endocytosis, but only P57–68 enter the cells by lipid raft/caveolae-mediated endocytosis. Zimmermann et al. also confirmed the effect of M-β-CD on the entrance of P31–43 and P57–68-fluorescence labeled in CaCo-2 cells [8]. The fact that a precise way of the entrance of these peptides has been discovered opens several new opportunity of finding drugs that can interfere with the entrance of the indigested gliadin peptides. These differences in the route of entrance have to be taken into account for future drug interference. Still not answered is the question how these peptides enter the cells. A receptor for both gliadin peptides has been looked for but not found [8]. This is not entirely surprising as many bioactive peptides in nature do not need a receptor to enter the cells, as they can interact with the membranes directly [9]. Noteworthy, P31–43, but not P57–68, can interact with a membrane mimetic environment [10].

### Localization of gliadin peptides in the endocytic vesicles

Next, the localization of gliadin peptides in intestinal epithelial cells has been investigated. Interestingly, both gliadin peptides P31–43 and P57–68-liss have been localized in the early compartment of endocytosis in CaCo-2 cells after 30-min incubation, but only 3 h after treatment, there is segregation of P41–43/49, but not of P57–68, in the early endosomal compartment. This interesting segregation of P31–43 in the early compartment has been demonstrated by different groups and by different methods, both in epithelial cells in culture and in intestinal biopsies of CD patients and controls in ex vivo experiments [11–14].

### Biological consequences of P31–43/49 segregation in the early endocytic compartment

The biological consequences of this segregation can be several and in different pathways. In fact, P31–49, unlike P57–67, bypassing HLA-DR-positive late vesicles and escaping antigen presentation at the basolateral membrane cannot stimulate gluten-sensitive T cells [11], preventing oral tolerance.

Moreover, the segregation in the early compartment of P31–43 peptide is due to a direct effect of the peptide on the endocytic trafficking. In fact, P31–43 is strikingly similar to a region of hepatocyte growth factor-regulated substrate (HRS) kinase, a key molecule regulating endocytic maturation, which is localized on the membranes of early endocytic vesicles [13].

The sequence similarity between gliadin peptide P31–43 and HRS is located in a small area of the proline/glutamine-rich domain of HRS. The COOH-terminal of HRS contains a clathrin-binding domain that binds clathrin to clathrin-coated vesicles and is one of the domains needed to localize HRS to the vesicle membranes. Both in CaCo-2 cells and in the celiac enterocytes, P31–43 localizes in the early endosomes and delays vesicular trafficking of several different cargos [13]. Endocytosis has many effects on signaling; in fact, signaling pathways and endocytic pathways are regulated in a reciprocal manner. Consequently, endocytosis affects several cell functions, ranging from proliferation to actin organization, cell motility, and stress/innate immunity activation. Gliadin peptide P31–43 can increase IL-15, one of the major effectors of innate immunity in CD on CaCo-2 cell surface, not affecting mRNA or protein levels, but increasing the recycling of the complex IL-15/IL-15 receptor alpha to the membrane [4, 15, 16]. Biological effects of gliadin peptide P31–43 have been recently reviewed [4]. Notably, impairment of the protein targeting and function has been described also when Frazer’s fraction of gliadin has been used, due to an interaction with the actin cytoskeleton [17, 18].

The block in the early compartment of gliadin peptides P31–43 or 49 can be overcome by cholera toxin B [12], by CD patients serum [19], or by linking the peptide to biotin [20]. Strikingly, even when forced into the late compartment by biotin, for example, this peptide has some important biological effects on cellular stress and inflammation [20].

Other composts such as antibody against tissue transglutaminase (tTG) [7] or probiotics [21] can prevent gliadin peptides entrance in the cells, with reduced biological effects for several read outs, indicating that their entrance is crucial to the activation of cellular signaling. All together, these observations indicate that gliadin peptides P31–43/49 have important biological effects by interfering with the endocytic pathway. These data suggest that indigested gliadin peptides have strong biological activity not only by activating the adaptive immune response but also by initiating at the level of the intestinal epithelium stress/innate immune response.

### Gliadin peptides transcytosis

Few studies are available on the destiny of gliadin peptides after they enter the cells in intestinal biopsies. Among these, Ménard et al. have established that P31–49 and 33 mer do enter the epithelial cell of intestinal biopsies from CD patients by intracellular pathway, excluding the paracellular entrance [22]. Lebreton et al. have shown that in active celiac mucosa, gliadin peptides can be transcytosed from the apical of the intestinal epithelium to the basolateral side together with transferrin and IgA avoiding the late endocytic compartment. Transcytosis of IgA and transferrin is a well-known phenomenon, and transferrin increases in proliferating cells. The authors observe that this transcytosis can be prevented by antibodies against tTG and happens only when IgA–antigliadin and transferrin increase in the intestinal mucosa, basically when the disease is already in the active phase [23]. Moreover, Schumann et al. demonstrated that transcytosis of 33-mer is Ras-related in brain **(**Rab 5) dependent (Rab 5 is a marker of the early endocytic compartment) and higher in active CD, than in patients on a gluten-free-diet and in controls [15]. What happens to transcytosis of gliadin peptides before the disease is manifested is not known. On the other side, transcytosis of gliadin peptides in in vitro systems such as CaCo-2 cells strongly depends on the length of the peptides and integrity of the epithelial barrier [5, 6, 8]. Moreover, these observations decidedly exclude the possibility that gliadin peptides enter by a paracellular pathway and, although these data have been published several years now, this notion is still pointlessly debated in the field.

### In vivo findings

In vivo findings about viability of gliadin peptides are very scarce. One interesting paper on the detection of gliadin peptide 33 mer in the feces of CD patients on a GFD fed with known amount of gliadin [24] indicates that 33-mer gliadin peptide is undigested by the intestinal brush border in vivo. Moreover, in a monkey model of gluten sensitivity, 33-mer peptide can be detected in the serum when the disease starts, indicating that this peptide can trespass the mucosa intact in vivo [25]. No reports are present in the literature about the 25 mer (or 31–43/55) in vivo. The detection of indigested peptides in the feces can be used as a marker of diet complaint, a test that can be very useful in cases of doubt or for the monitoring of adolescent diet.

## Conclusions

Although the study of gliadin peptides trafficking is an important way to understand the mechanisms of CD, many questions still have to be answered mainly regarding the specificity of the effects of gliadin peptides in CD cells and intestinal epithelium. Why gliadin peptides are so toxic for celiac disease patients? Is there a constitutive defect in CD cells that renders them more sensitive to gliadin peptides effects? Are they toxic in control cells? Is prevention of entrance of gliadin peptides in the epithelium enough to prevent the disease? Future study will be able to answer these questions.

## Abbreviations

BBM: brush border membranes
Ca++: calcium
CD: celiac disease
CD4: cluster of differentiation 4
HLA: human leucocytes antigen
HRS: hepatocyte growth factor-regulated substrate
IgA: immunoglobulin A
IL15: interleukin-15
liss: lissamine
LP: lamina propria
mRNA: messenger ribonucleic acid
M-β-CD: methyl-β-cyclodextrin
Rab 5: Ras-related in brain
